# Supplementary material for: Diagnostic Performance of AFP, AFP-L3, or PIVKA-II for Hepatitis C Virus-Associated Hepatocellular Carcinoma: A Multicenter Analysis
Source: J Clin Med. 2022 Aug 29;11(17):5075. doi: 10.3390/jcm11175075 (PMC9456633; doi:10.3390/jcm11175075)

Journal of
Clinical Medicine

Supplement Table S1. Three serum biomarkers alone and in combination for the detection of HCV-HCC among patients with HCV-cirrhosis.

	AUC (95% CI)	Clinical Cut-Off Value	Sensitivity (%) (95% CI)	Specificity (%) (95% CI)	PPV (%) (95% CI)	NPV (%) (95% CI)	Positive LR	Negative LR
**AFP**	0.802 (0.743–0.853)	20 ng/mL	56.2 (45.3–66.7)	87.4 (80.3–92.6)	75.8 (65.6–83.7)	74.0 (69.0–78.4)	4.2	0.6
**PIVKA-II**	0.900 (0.852–0.937)	40 mAU/mL	78.7 (68.7–86.6)	89.0 (82.2–93.8)	83.3 (75.1–89.2)	85.6 (79.9–89.9)	7.1	0.2
**AFP-L3**	0.681 (0.614–0.742)	10%	48.2 (38.1–59.1)	82.7 (75.0-88.8)	60.7 (49.3–71.1)	65.6 (61.4–79.6)	2.4	0.7
**AFP+PIVKA-II**	0.931 (0.888–0.961)		87.6 (79.0–93.7)	89.8 (83.1–94.4)	85.7 (78.1–91.0)	91.2 (85.6–94.8)	9.2	0.2
**PIVKA-II+ AFP-L3**	0.901 (0.853–0.937)		86.5 (77.6–92.8)	85.8 (78.5–91.4)	81.1 (73.5–86.9)	90.1 (84.2–93.9)	6.1	0.2
**AFP+ AFP-L3**	0.780 (0.719–0.834)		70.8 (60.2–79.9)	78.7 (70.6–85.5)	70.0 (61.9–77.0)	79.4 (73.3–84.3)	3.3	0.4
**AFP+PIVKA-II+AFP-L3**	0.928 (0.885–0.959)		86.5 (77.6–92.8)	92.9 (87.0–96.7)	89.5 (81.9–94.2)	90.8 (85.3–94.3)	12.2	0.2

AFP, alpha-fetoprotein; AFP-L3, lens culinaris agglutinin A-reactive fraction of alpha-fetoprotein; AUC, area under curve; HCC, hepatocellular carcinoma; HCV, hepatitis C virus;
LR, likelihood ratio; NPV, negative prediction value; PIVKA-II, protein induced by vitamin K absence or antagonist-II; PPV, positive prediction value.

Supplement Table S2. Clinical characteristics of early-stage HCV-HCC.

N (%)	BCLC Stage 0/A (N = 57)	8th TNM Stage I (N = 57)
**Baseline characteristics**		
**Age, years***	57.0 ± 10.9	59.0 ± 11.0
**Male sex**	45 (78.9)	43 (75.4)
**Child-Pugh grade**		
**A**	55 (96.5)	54 (94.7)
**B+C**	2 (3.5)	3 (5.3)
**Cirrhosis**	47 (82.5)	49(85.9)
**Platelet, ×10^9/L***	148 (92, 180)	147 (89, 180)
**Bilirubin, µmol/L***	14.7 (11.9, 21.6)	16.7 (13.9, 23.6)
**Albumin, g/L***	44.9 ± 5.4	44.8 ± 5.4
**Tumor characteristics**		
**Tumor size, cm***	4.1 (2.5, 7.0)	4.1 (2.6, 7.0)
**≥3 cm**	31 (54.4)	26(45.6)
**Multiple tumors**	10 (17.5)	15 (26.3)

*Values are mean ± standard deviation or median with interquartile range.

AFP, alpha-fetoprotein; AFP-L3, lens culinaris agglutinin A-reactive fraction of alpha-fetoprotein; BCLC, Barcelona Clinic Liver Cancer; HCC, hepatocellular carcinoma; HCV, hepatitis C virus; PIVKA-II, protein induced by vitamin K absence or antagonist-II; TNM, tumor node metastasis

Supplement Figure S1. Diagnostic performance of AFP, PIVKA-II, or AFP-L3 alone and in combination for the detection of HCV-HCC among patients with cirrhosis. (A) AFP, PIVKA-II, or AFP-L3 alone; (B) Any combination of two or three biomarkers; for the detection of early-stage HCV-HCC (BCLC stage 0-A) AFP, alpha-fetoprotein; AFP-L3, lens culinaris agglutinin A-reactive fraction of alpha-fetoprotein; AUC, area under the curve; HCC, hepatocellular carcinoma; HCV, hepatitis C virus; PIVKA-II, protein induced by vitamin K absence or antagonist-II.

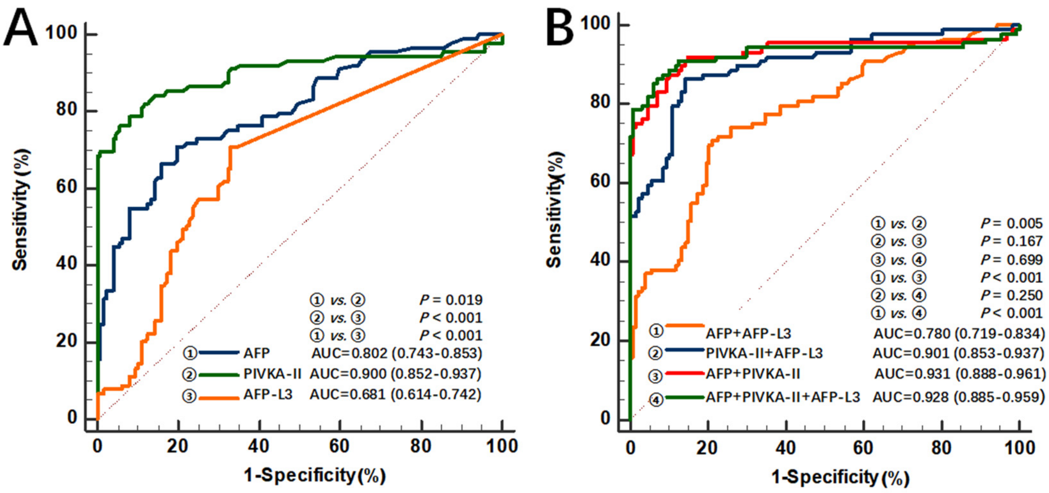

Supplement: Supplementary file 1 [file jcm-11-05075-s001.zip › jcm-1859861-supplementary.pdf]
